# The global pediatric antiretroviral market: analyses of product availability and utilization reveal challenges for development of pediatric formulations and HIV/AIDS treatment in children

**DOI:** 10.1186/1471-2431-10-74

**Published:** 2010-10-17

**Authors:** Brenda Waning, Ellen Diedrichsen, Elodie Jambert, Till Bärnighausen, Yun Li, Mieke Pouw, Suerie Moon

**Affiliations:** 1 Boston University School of Medicine, Department of Family Medicine, One Boston Medical Center Place, Dowling 5 South, Boston, MA 02118, USA; 2 Utrecht University, Utrecht, Netherlands; 3 Médecins Sans Frontières, Geneva, Switzerland; 4 Harvard School of Public Health, Department of Global Health and Population, Boston, MA 02115, USA; 5 University of KwaZulu-Natal, Africa Centre for Health and Population Studies, 3935 Mtubatuba, KZN, South Africa; 6 Sustainability Science Program, Center for International Development, Harvard Kennedy School of Government, Cambridge, MA 02138, USA

## Abstract

**Background:**

Important advances in the development and production of quality-certified pediatric antiretroviral (ARV) formulations have recently been made despite significant market disincentives for manufacturers. This progress resulted from lobbying and innovative interventions from HIV/AIDS activists, civil society organizations, and international organizations. Research on uptake and dispersion of these improved products across countries and international organizations has not been conducted but is needed to inform next steps towards improving child health.

**Methods:**

We used information from the World Health Organization Prequalification Programme and the United States Food and Drug Administration to describe trends in quality-certification of pediatric formulations and used 7,989 donor-funded, pediatric ARV purchase transactions from 2002-2009 to measure uptake and dispersion of new pediatric ARV formulations across countries and programs. Prices for new pediatric ARV formulations were compared to alternative dosage forms.

**Results:**

Fewer ARV options exist for HIV/AIDS treatment in children than adults. Before 2005, most pediatric ARVs were produced by innovator companies in single-component solid and liquid forms. Five 2-in1 and four 3-in-1 generic pediatric fixed-dose combinations (FDCs) in solid and dispersible forms have been quality-certified since 2005. Most (67%) of these were produced by one quality-certified manufacturer. Uptake of new pediatric FDCs outside of UNITAID is low. UNITAID accounted for 97-100% of 2008-2009 market volume. In total, 33 and 34 countries reported solid or dispersible FDC purchases in 2008 and 2009, respectively, but most purchases were made through UNITAID. Only three Global Fund country recipients reported purchase of these FDCs in 2008. Prices for pediatric FDCs were considerably lower than liquids but typically higher than half of an adult FDC.

**Conclusion:**

Pediatric ARV markets are more fragile than adult markets. Ensuring a long-term supply of quality, well-adapted ARVs for children requires ongoing monitoring and improved understanding of global pediatric markets, including country-based research to explain and address low uptake of new, improved formulations. Continued innovation in pediatric ARV development may be threatened by outdated procurement practices failing to connect clinicians making prescribing decisions, supply chain staff dealing with logistics, donors, international organizations, and pharmaceutical manufacturers. Perceptions of global demand must be better informed by accurate estimates of actual country-level demand.

## Background

Accessing quality treatment and care remains an uphill battle for families of children living with HIV/AIDS in resource-poor settings. For many years, the lack of easy-to-use pediatric formulations for some antiretroviral (ARV) medicines and the high costs of others hindered efforts to deliver medical care to this vulnerable population [1].

From an industry perspective, the disincentives to develop and produce pediatric ARVs are numerous and powerful. Pediatric ARV markets are always smaller and less attractive than adult markets. In the United States and Europe, HIV infections in infants and young children have been nearly eliminated [2], leaving little demand for pediatric ARV formulations in these markets.

In order to develop new pediatric dosage forms for use in developing countries with larger pediatric ARV demand, additional research must first be conducted in children, including costly clinical trials, bioequivalence, bioavailability, dose-ranging, and pharmacokinetic studies [3,4]. The implementation of comprehensive services to prevent mother-to-child transmission (PMTCT) of HIV remains low in many countries [5]; however, if recent initiatives to reduce vertical HIV transmission are successful [6], pediatric antiretroviral demand will further diminish, reducing any returns on investment for developing pediatric ARVs. After development, the per-unit production costs of pediatric ARVs are likely high because small quantities impede the realization of economies of scale in production and distribution [3].

Further compounding these disincentives are the innate complexities of pediatric formulation markets. Numerous products are needed in varying strengths to accommodate changing doses as children grow, which fragments the pediatric market for a given ARV into even smaller niches. Moreover, as children move through infancy, toddler, and childhood stages, the optimal dosage form changes as well. Liquids (syrups, suspensions, and solutions) are needed to treat infants but pose logistical challenges: many need refrigeration and, because of large bottle sizes and heavy weight, are difficult for families to carry home. In low resource settings, measuring and delivering the correct liquid doses can also be challenging. Powders and dispersible tablets that can be mixed with water are an option, but they require access to clean water and often have unpleasant tastes that are unacceptable to infants. As children get older and the necessary volumes of liquid ARVs become too large, they require other products, such as chewable tablets and sprinkles, until they reach an age when they can swallow solid tablets [7].

Despite these market disincentives for pharmaceutical manufacturers, fortunately, important advances have recently been made in the development and production of pediatric ARV formulations quality-certified by the World Health Organization (WHO) Prequalification Progamme [8], the United States (US) Food and Drug Administration (FDA) [9,10], or other stringent regulatory authorities. This progress can be credited to persistent lobbying and innovative interventions from HIV/AIDS activists, civil society organizations, and international organizations.

Médecins Sans Frontières, for example, has consistently drawn attention to the particularly glaring neglect of children in HIV/AIDS treatment programs and suggested that the lack of child-friendly versions of ARVs contributes to high rates of HIV/AIDS deaths in children under two years of age [11,12]. In November 2004, the United Nations Children's Fund (UNICEF) and WHO held a technical consultation on improving access to appropriate pediatric ARV formulations during which experts identified missing pediatric formulations considered to be high priority and discussed ways to galvanize pharmaceutical companies to produce them [7]. Shortly thereafter, two global initiatives were launched. *Unite for Children, Unite Against AIDS*, was begun by UNAIDS, UNICEF, and others in 2005 as a platform for all partners engaged in pediatric HIV/AIDS programs [13]. The first *WHO Model List of Essential Medicines for Children *was released in 2007 [14] and *Make medicines child size*, led by WHO, was launched in late 2007 to raise awareness and improve access to medicines that are safe for children under age 12 [15].

On the implementation side, both the United States' President's Emergency Plan for AIDS Relief (PEPFAR) [16] and UNITAID [17] made commitments to prioritize the needs of children. The Clinton Health Access Initiative (CHAI) [18] and the Supply Chain Management System (SCMS) [19] conduct large-scale purchasing on behalf of UNITAID and PEPFAR, respectively. Given the substantial overlap of funding in some countries by UNITAID, the Global Fund to Fight AIDS, Tuberculosis, and Malaria (GFATM) [20], and PEPFAR, agreements were made with countries and major donors that UNITAID would initially be the primary source of funding for pediatric ARVs (D Jamieson, SCMS, personal communication) in those countries. Such coordination allows for optimization of resources and avoidance of service duplication. In countries where UNITAID and PEPFAR are not active, GFATM-supported HIV/AIDS programs procure their ARVs independently.

The will of these international organizations to invest in pediatric antiretroviral therapy (ART) created incentives for producers to enter the market, as manufacturers could be relatively certain of a minimum volume of purchases from reliable clients [21,22]. The ensuing scale-up of ARV delivery to children in developing countries then progressed dramatically. Whereas only 10% of children in need were being treated in 2005, 38% were receiving ART by the end of 2008 [5]. However, despite these advances in product development and treatment coverage, the literature suggests that retaining children in HIV/AIDS programs remains problematic [23,24]. In 2009 the Clinton Foundation reported infant losses to follow-up of 32% (in Cameroon) and 53% (in an eight-country meta-analysis) [25].

Clearly, critical challenges remain. Three out of five children needing ART are not receiving it [5]. A preliminary examination of ARV purchase data suggests that many countries are not yet using new, improved pediatric formulations [26,27]. Finally, there are a number of ARVs for which appropriate pediatric formulations are still not available. According to Médecins Sans Frontières, appropriate pediatric formulations are still lacking for a range of important ARVs, including efavirenz, darunavir and other ritonavir-boosted protease inhibitors (in addition to lopinavir/ritonavir) [28].

Most pediatric fixed-dose combinations (FDCs) developed to date have included stavudine and zidovudine. Aside from low demand, few barriers existed for the development of these products. A substantial amount of research had already been conducted on these ARVs in children, patents were generally absent or unenforced [29], and manufacturers had lots of experience producing adult versions. In contrast, for newer ARVs, little research has been conducted in children, patent barriers are more widespread, and manufacturers have less experience producing adult FDCs containing these ARVs. To ensure that companies develop pediatric versions of these medicines, a better understanding is needed of both the supply and the demand side of the pediatric ARV market. However, to date, no research has been published on the characteristics or the evolution of this market.

In order to fill this knowledge gap, this paper examines trends in the availability of WHO-recommended ARVs in quality-certified pediatric formulations, and describes the rate and extent of product uptake across developing countries and among international donors to guide next steps towards improving child health.

## Methods

**Figure 1:**
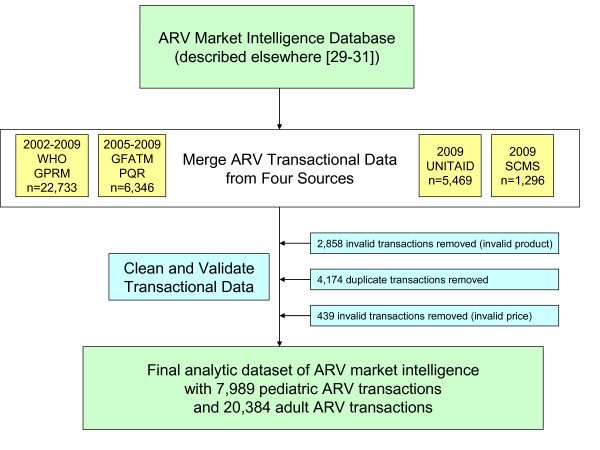
**Data overview**.

We utilized an ARV market intelligence database comprised of data from multiple sources, including product approvals by the US FDA [9,10] and certifications by the WHO Prequalification Progamme [8]. This database is described in more detail elsewhere [30-32]. Into it we merged 2009 transactional data of donor-funded ARV purchases provided directly to researchers by the Clinton Health Access Initiative (CHAI) [18] on behalf of UNITAID [17] and the Supply Chain Management System (SCMS) [19] on behalf of PEPFAR [16] as well as publicly posted transactions in the WHO *Global Price Reporting Mechanism *[27] and the GFATM *Price Quality Report *[26] from 2002-2009. Information and algorithms in the market intelligence database were used to clean and validate ARV transactional data, which was then limited to purchases made for ARV formulations predominantly used in children (Figure 1).

We examined trends in quality-certification of pediatric ARVs by WHO [8] and the FDA [9,10] in relation to treatment regimens recommended by WHO for infants and children [33,34]. We also described purchase trends for pediatric ARV formulations (liquid, solid, dispersible), including numbers of purchasing countries, from 2004 to 2009. A solid product is defined as a medicine intended to be swallowed, while a dispersible ARV tablet dissolves when placed in a small amount of water. Liquids are syrups, solutions, and suspensions. We differentiate single-component ARVs from FDC dosage forms with the labels "single" and "FDC". We describe FDC products using a "/" between ARVs included in a given FDC and use the terms brand and innovator interchangeably to denote the initial developer of a medicine.

We calculated trends in purchaser (GFATM, SCMS, UNITAID) market share by value for brand and generic pediatric dosage forms from 2002 to 2009. For FDC versions of pediatric ARVs, we provided percent purchaser market share by volume for 2008 and 2009.

Price comparisons of ARV dosage forms (pediatric FDC, liquid, and adult FDC) were calculated using prices paid by CHAI/UNITAID based upon WHO-recommended doses [33,34] and presented as price per person per year in United States dollars (USD). All ARV prices provided by GFATM, WHO, CHAI, and SCMS in USD were adjusted to the January-December 2009 time period using the annual US Consumer Price Index [35].

## Results

### Priority pediatric ARVs: WHO recommendations, production and purchase trends

In 2007, the WHO Paediatric Antiretroviral Working Group identified a total of 40 priority pediatric ARV products (19 urgent, 10 high, and 11 important) for pediatric HIV/AIDS treatment (Table 1) [33]. Only 17 of the 40 pediatric ARV products were categorized by WHO as "ideal" dosage forms. Sixteen of the 40 recommended ARV products were actually produced and purchased by countries for pediatric HIV/AIDS treatment. Some new pediatric products originally produced and purchased are no longer in demand because subsequent changes in dosing guidelines meant the new formulations no longer matched the revised dosing recommendations. In 2009, the WHO Expert Committee on the Selection and Use of Essential Medicines revised the list of priority pediatric ARVs to include 17 of the 18 ARVs originally categorized as ideal in 2007 (Table 1) plus one new formulation (ABC60/NVP50/ZDV60) and one new ARV (atazanavir) [36].

**Table 1:** Priority pediatric ARV formulations

Pediatric ARV Dosage Form	2007 WHO Recommendation	Ideal Dosage Form	Produced and purchased	2009 WHO Recommendation
3TC30/NVP50/ZDV60	Urgent	yes	yes	yes

3TC30/NVP60/ZDV60	Urgent			

3TC75/NVP100/ZDV150	Urgent			

3TC30/ZDV60	Urgent	yes	yes	yes

3TC75/ZDV150	Urgent			

3TC30/d4T6	Urgent	yes	yes	yes

3TC75/d4T15	Urgent			

3TC30/NVP50/d4T6	Urgent	yes	yes	yes

3TC20/NVP35/d4T5	Urgent		yes	

3TC30/NVP50/d4T7	Urgent			

3TC60/NVP100/d4T12	Urgent		yes	

3TC40/NVP70/d4T10	Urgent		yes	

NVP 50	Urgent	yes		yes

NVP 100	Urgent			

LPV100/r25	Urgent	yes	yes	yes

LPV90/r22.5	Urgent			

ABC 60	Urgent	yes	yes	yes

ABC 120	Urgent			

ABC 150	Urgent			

EFV 100	High	yes	yes	yes

EFV 600	High		yes	

ABC60/3TC30	High	yes	yes	yes

ABC150/3TC75	High			

ZDV 60	High	yes		yes

ZDV 100	High		yes	

ABC60/3TC30/ZDV60	High	yes		yes

ABC150/3TC75/ZDV150	High			

d4T 6	High	yes		yes

d4T 15	High			

ddI 125	Important		yes	

ddI 200	Important		yes	

3TC 30	Important	yes		yes

3TC 75	Important			

3TC 150	Important		yes	

EFV100/FTC35	Important	yes		yes

FTC 35	Important	yes		yes

RTV 25	Important	yes		yes

RTV 100	Important			

FPV*	Important	yes		yes

DRV*	Important			

ABC60/NVP50/ZDV60				yes

ATV*				yes

*dose to be determined

### Overview of FDA-approved and WHO-prequalified pediatric ARVs

**Figure 2:**
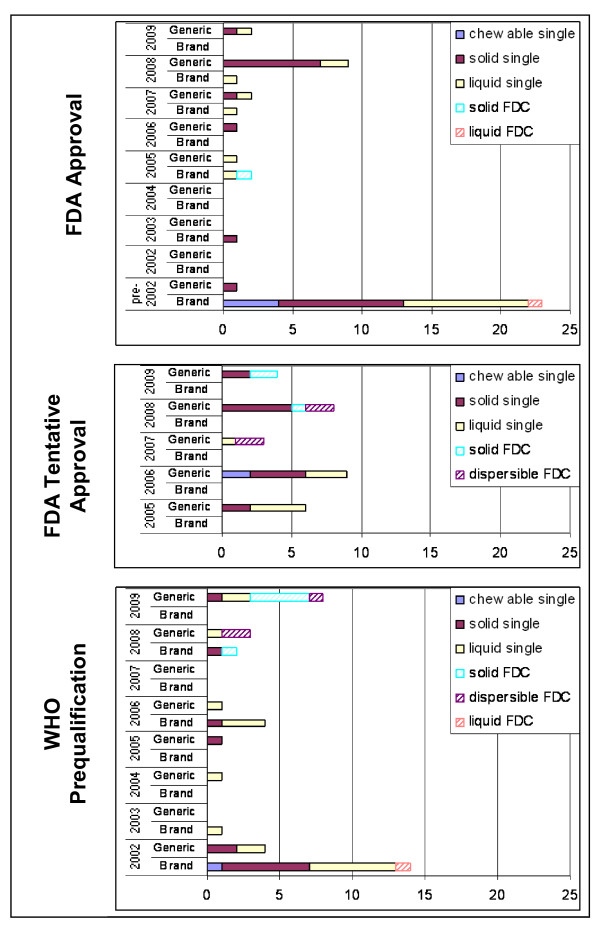
Trends in innovator and generic pediatric ARV formulations certified by WHO and FDA*. *Includes all pediatric ARV approvals for all manufacturers; overlap between FDA approval and WHO prequalification exists as some products are certified by both organizations.

Prior to 2002, 23 of 24 (96%) FDA-approved pediatric formulations were produced by innovator companies (Figure 2), reflecting demand from patent-protected markets in the US and Europe. The establishment of the GFATM in 2002 created instantaneous demand for affordable ARVs in developing countries, many of which overcame intellectual property barriers to purchase low-cost generic medicines [29]. Eighteen pediatric ARVs (14 innovator and four generic) were certified by WHO in 2002, the first year of the program, but only three formulations were pre-qualified in the following three years.

Over the entire time period a total of 113 ARV formulations produced by eight innovator and eight generic manufacturers were certified. Most innovator ARVs were approved before 2005 and most generic ARVs were approved in 2005 or later. Examination of all certifications by dosage form reveals 44 liquid, 55 solid, seven chewable, and seven dispersible products. All dispersible products were generic and certified in 2007 or later.

**Figure 3:**
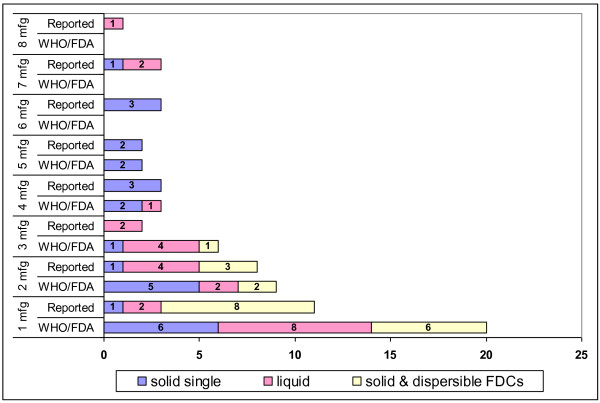
**Number of manufacturers certified and reported to supply each pediatric ARV**.

For the majority of pediatric FDCs, only one manufacturer is quality-certified by either the WHO or the FDA. Six of nine (67%) solid and dispersible FDCs are produced by only one quality-certified manufacturer (Figure 3). Similar patterns exist for other pediatric ARV dosage forms with six solid single-component ARVs and eight liquid ARVs supplied by only one quality-certified producer.

Among pediatric ARVs purchased and reported (both quality-certified and non-quality certified), eight solid and dispersible FDCs were supplied by only one manufacturer (Figure 3). Many single component solid ARVs and liquid ARVs, however, were supplied by two or more manufacturers.

The 2002 WHO first-line treatment guidelines for infants and children included five ARVs and three regimens [37] (Table 2). In 2006, the WHO revised their guidelines to include six ARVs and six preferred first-line regimens [34]. Approximately 68% of all WHO and FDA product certifications were for ARVs recommended by WHO in first-line regimens. Four and six preferred second-line regimens, all of which contain didanosine and a protease inhibitor, were listed on WHO 2002 and 2006 guidelines, respectively. A 2008 guidance by WHO listed three first-line regimens as well as three regimens for infants exposed to certain ARVs

**Table 2:** WHO-recommended regimens for infants and children

Year of WHO Guideline	First-Line Regimen	Second-Line Regimen
2002	ZDV + 3TC + ABC	d4T + ddI + PI* or d4T + ddI + (EFV or NVP)

	ZDV + 3TC + (NVP or EFV)	d4T + ddI + PI*

2006 Preferred	(ZDV or d4T) + 3TC + (NVP or EFV)	ddI + ABC + PI**

	ABC + 3TC + (NVP or EFV)	ddI + ZDV + PI**

2006 Alternative	(ZDV or d4T) + 3TC + ABC	ddI + (EFV or NVP) +PI**

2008***	(ZDV or d4T or ABC) + 3TC + NVP	

	(ZDV or d4T or ABC) + 3TC + LPV/r	

*PI options include LPV/r and NFV

**PI options include LPV/r, SQV/r, and NFV

***Infants <12 months of age

Because limited research has been conducted in children with HIV/AIDS, fewer ARV treatment options exist for infant and children as compared to adults. This lack of pediatric research is particularly relevant for newer ARVs. Whereas tenofovir is now recommended by the WHO for first-line treatment of adolescents and adults [38,39] and is being widely adopted by countries, tenofovir is not recommended for use in infants and children due to insufficient research on safety and toxicity [34].

Because of interactions between nevirapine and rifampicin (anti-tuberculosis medicine), the WHO recommends use of efavirenz in place of nevirapine for HIV/AIDS in adults with tuberculosis co-infection [38]; however no data is available on safety and efficacy of efavirenz in children under three years of age [34].

Pediatric HIV/AIDS treatment options are further reduced if newborns were exposed to single dose nevirapine for PMTCT or maternal non-nucleoside reverse transcriptase inhibitor (NNRTI) therapy. When protease-inhibitors are used for first-line treatment in infants with NVP and/or NNRTI maternal exposure, infants and children are left with few ARV options for second-line treatment [40]. Whereas boosted darunavir, etravirine, and raltegravir are potential options in adults who fail protease inhibitor regimens [39], none of these options are available in pediatric formulations and little research on use of these ARVs in children has been conducted.

Only one pediatric ARV FDC (LPV/r) existed before the establishment of the GFATM and it was only available in a liquid form requiring refrigeration. The first pediatric 3-in-1 FDC to accommodate WHO-recommended first line regimens was quality-certified in 2007 (Table 3), lagging four years behind the first adult version.

**Table 3:** Initial quality certification of FDC pediatric ARVs*

	2000	2005	2007	2008	2009
**2-in-1 FDCs**					

ABC60/3TC30				solid	

3TC30/d4T6				dispersible	

3TC60/D4T12				dispersible	

3TC30/ZDV60					solid

LPV100/RTV25		solid			

LPV80/RTV20 per ml	liquid				

**3-in-1 FDCs**					

ABC60/3TC30/ZDV60					solid

3TC30/NVP50/d4T6			dispersible		

3TC60/NVP100/d4T12			dispersible		

3TC30/NVP50/ZDV60					dispersible

*recommended on 2006 WHO pediatric HIV/AIDS treatment guidelines

Since 2005, a total of five 2-in-1 pediatric FDCs and four 3-in-1 pediatric FDCs had been FDA-approved and/or WHO-prequalified. Eight of these nine (89%) FDCs support first-line regimens. The first pediatric heat-stable, ritonavir-boosted protease inhibitor (LPV/r) was certified in 2005 and remains the only FDC available to support second-line treatment in children. The first dispersible tablets were approved in 2005 with five FDCs available by the end of 2009.

### Purchase trends and market share for pediatric ARVs

**Figure 4:**
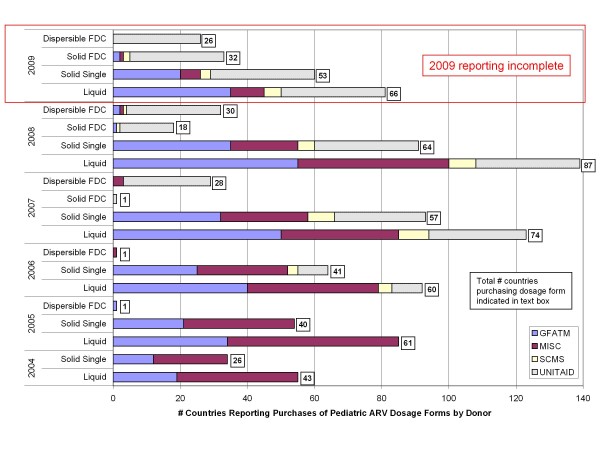
Country pediatric ARV purchase trends, by purchaser, 2004-2009*. *total number of unique countries purchasing ARV dosage form is indicated in text boxes (some overlap of countries across some donor programs).

Few countries purchased pediatric ARVs before 2004. The number of countries purchasing liquid and solid single-component pediatric ARVs increased steadily from 43 and 26, respectively in 2004 to 85 and 64, respectively in 2008 (Figure 4). These liquid and solid single-component products were reported by large numbers of countries across GFATM, UNITAID, and Miscellaneous categories.

A total of 33 and 34 countries reported either solid or dispersible pediatric FDC purchases in 2008 and 2009, respectively. Pediatric FDC purchases, however, have been largely limited to countries supported by UNITAID, with 29 and 31 countries reporting FDC purchases (solid and dispersible) in 2008 and 2009, respectively. Only three GFATM countries reported pediatric FDC ARV purchases in 2008, while SCMS reported pediatric FDC purchases for only two countries in 2008 and 2009.

Looking more closely at purchase trends for different FDC dosage forms, purchases for dispersible FDCs were first reported in 2005 but increased sharply in 2007 with 28 countries reporting purchase transactions. UNITAID accounted for 26 of the 28 (93%) countries reporting dispersible FDC purchases. This trend continues through 2009 when UNITAD reported dispersible FDC purchases in 26 countries and no other purchases were reported outside of UNITAID.

Similar purchase trends are noted for solid pediatric FDCs whereby UNITAID reported purchase transactions for 16 and 28 countries in 2008 and 2009, respectively, while very few countries outside of UNITAID reported solid FDC transactions.

**Figure 5:**
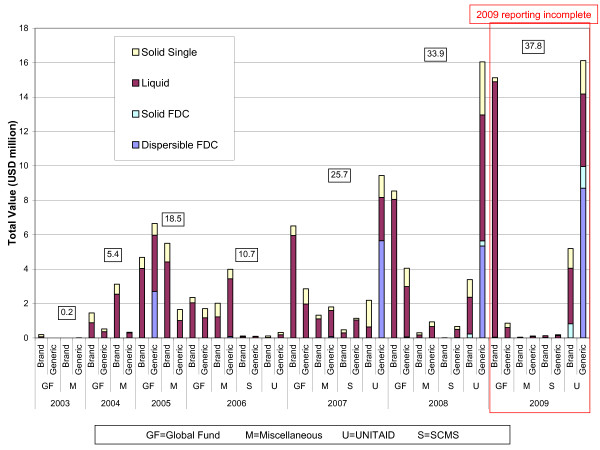
**Pediatric ARV market trends (value), 2003-2009**.

The total donor-funded pediatric ARV market increased from approximately US $5 million in 2004 to $34 million in 2008, with total 2009 purchases likely to be more than $40 million once reporting is complete (Figure 5). While the pediatric ARV market has grown rapidly, its current size is a small fraction of the US $500 million reported thus far in 2009 for adult ARV solid dosage forms.

From 2007 to 2009 UNITAID accounted for 62-93% of generic purchases, while the GFATM accounted for 62-74% of all innovator purchases. Careful examination of 2009 GFATM brand purchases reveals that the Russian Federation and South Africa account for 80% and 14% of all GFATM branded spending, respectively, and 59% and 10% of branded spending, respectively, of all donor purchases. The Russian Federation purchased five branded liquids (ZDV, NVP, 3TC, ddI, ABC) and South Africa purchased one branded liquid (LPV/r). The remaining GFATM countries account for only 6% of 2009 GFATM branded ARV purchases.

**Figure 6:**
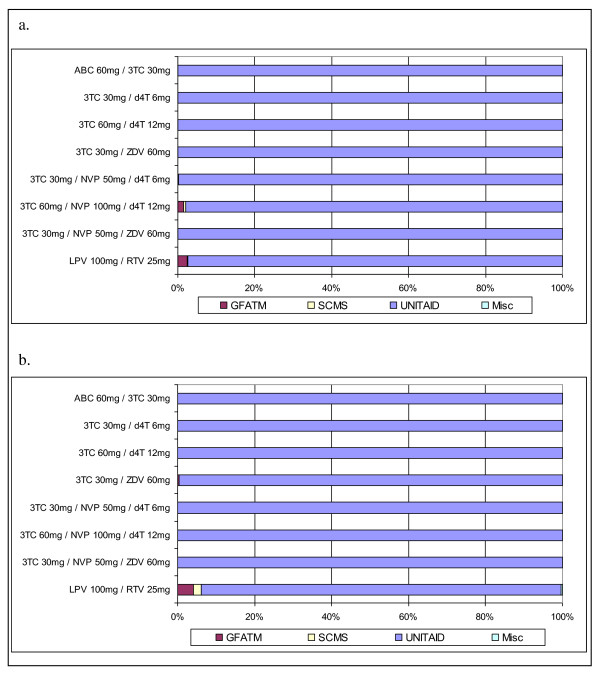
**Purchaser market share (volume) for solid & dispersible pediatric FDCs, 2008 and 2009**. a. Pediatric FDC ARV Market Share (volume) by purchaser, 2008. b. Pediatric FDC ARV Market Share (volume) by purchaser, 2009.

Further examination quantifies the low uptake of dispersible and solid pediatric FDCs outside of UNITAID. In 2008, UNITAID accounted for 100% of market volume for five of eight FDCs and 97-99% of market volume for the remaining three FDCs (Figure 6a). UNITAID held similar pediatric FDC market dominance in 2009 (Figure 6b).

### Price comparisons of pediatric ARV formulations

Prices for all pediatric ARV formulations continue to drop, with FDCs remaining consistently less expensive than liquid formulations (Table 4). Liquid alternatives were 2.3-3.2 times more expensive than dispersible products for stavudine-based FDCs in 2009 and ranged from 1.4-1.6 times more expensive than solid versions of zidovudine- and abacavir-based FDCs.

**Table 4:** Price comparison of ARV dosage forms, 2008-2009*

		Pediatric Defined Daily Dose			2008 Price/Person/Year (USD)			2009 Price/Person/Year (USD)		
	**Weight** **(kg)**	**Liquid** **(ml)**	**Pedi** **FDC** **(tab)**	**Adult** **FDC** **(tab)**	**Liquid**	**Pedi** **FDC**	**Adult** **FDC**	**Liquid**	**Pedi** **FDC**	**Adult** **FDC**

**2-in-1 FDCs**										

ABC60/3TC30	5	6/6	2		175	-		142	89	

3TC30/d4T6**	5	6/12	2		69	27		52	23	

3TC60/D4T12**	10	12/24	2	1	139	50	29	105	40	24

3TC30/ZDV60	5	6/12	2		71	44		56	40	

LPV100/RTV25	15	5	4	2	323	306	287	286	268	228

**3-in-1 FDCs**										

ABC60/3TC30/ZDV60	5	6/6/12	2		181	-		194	-	

3TC30/NVP50/d4T6**	5	6/10/12	2		122	33		83	29	

3TC60/NVP100/d4T12**	10	12/20/24	2	1	244	54	40	166	52	37

3TC30/NVP50/ZDV60**	5	6/10/12	2		123	56		86	53	

*based upon UNITAID prices reported by Clinton HIV/AIDS Initiative

**dispersible FDC

Some treatment programs treat children with half of an adult FDC once they reach weights of at least 10 kg. Prices for half of a stavudine-based adult FDC are 29-42% less expensive than the two stavudine-based pediatric FDCs.

## Discussion

In past years, great strides have been made in bringing to market quality-certified, pediatric ARV FDCs in dosage forms appropriate for use in low resource settings. Activists, international organizations and national governments have successfully lobbied for the development and production of pediatric ARV products. Five new 2-in-1 and four new 3-in-1 pediatric FDCs have been quality-certified by the WHO or FDA since 2005 in dosage forms appropriate for use in low resource settings. These new products include the first heat-stable, ritonavir-boosted protease inhibitor and five ARVs available as dispersible tablets, a formulation typically more acceptable to children than liquids and substantially less expensive, easier to store, less costly to distribute to health care facilities, and easier for care takers to carry home than liquid alternatives. The new solid and dispersible pediatric FDCs offer ease of administration and more reliable dosing than crushing or multiple-splitting of adult FDCs.

Despite these advantages, however, country purchases for ARVs in less desirable dosage formulations (liquid and solid, single-component ARVs) continue to show annual increases while uptake of the new solid and dispersible FDCs has been remarkably low outside of UNITAID-funded programs. UNITAID accounted for 97-100% of total market volume for all solid and dispersible pediatric FDCs purchased with donor-funds in both 2008 and 2009.

The GFATM provided funds to 116 countries for HIV/AIDS in 2008 [41] while UNITAID reported financing pediatric HIV/AIDS treatment in 44 countries by the end of 2009 [42]. The GFATM, SCMS, and UNITAID agreed that UNITAID would lead pediatric ARV procurement in countries they support, with UNITAID reporting pediatric ARV FDC purchases for 29 and 31 countries in 2008 and 2009, respectively. Only three and two countries from GFATM and SCMS, respectively, reported pediatric FDC ARV purchases in 2008. While donor coordination explains lack of FDC purchases in GFATM countries also receiving UNITAID funds, uptake of new pediatric FDCs in GFATM countries without UNITAID funding is remarkably low.

This study cannot explain the reasons for low uptake of improved pediatric formulations outside of UNITAID. Our results reveal the importance of additional operational research to identify barriers to product use. Still, we present some potential challenges at country level that may prevent or delay adoption of new products. To start, country-based staff may be unaware of recent developments and availability of new pediatric formulations. There may be reluctance to use new formulations, such as dispersible tablets, in regions where these types of medicines are not historically or currently used. Regulatory barriers, registration costs and difficulties, the need to revise treatment guidelines and the need to retrain all prescribers and caregivers may also contribute to under-utilization. Countries may be locked into long-term contracts that preclude them from switching to improved products or their demand may be too low to meet some suppliers' minimum purchase requirements.

To change from currently used ARVs to the new pediatric formulations may also produce challenges in supply chain management. Demand forecasting (*i.e*. determining the amount of medicine needed for country programs) is a challenging and complicated task [43] and insufficient focus has been placed on improving outdated procurement practices. It is possible that in the transition phase from one set of ARVs to another, the number of pediatric products in warehouses and on facility shelves increases substantially, making demand forecasting more complicated for some period of time. Thus, such transitions need to be carefully planned and monitored in order to avoid wastage and stock shortages.

It is also possible that the types of pediatric products created to date are not the products most desired at country level, or that practitioners and caregivers prefer to use half of an adult FDC instead of pediatric FDCs, when possible. Using adult FDCs for children in lieu of pediatric FDCs may simplify supply chain management of ARVs (procurement, storage, distribution, inventory management) as well as prescribing, dispensing, and administration by the caregiver. Lastly, countries may currently be in the process of transition and we are now observing a time lag between decisions to switch to newer products and actual implementation of those decisions.

While it is thus understandable for a number of reasons that the adoption of new, improved pediatric ARVs is a time-consuming process, such inertia may have undesirable side effects. For instance, it may falsely signal to pharmaceutical companies that the markets for improved pediatric ARVs are smaller than anticipated because of logistical and acceptability problems, deterring entry of new manufacturers and scale-up of production among existing ones [28].

International organizations and countries already face challenges obtaining existing pediatric ARV medicines. Reports that Bristol-Myers Squibb will encounter interruptions in the production of pediatric didanosine have created great concern for upwards of 7,000 children treated with this medicine [44,45]. Bristol-Myers Squibb is currently the only quality-certified producer of pediatric didanosine tablets and the amount of didanosine currently available may be insufficient to meet the needs of children on treatment during the period of supply interruption. Médecins Sans Frontières reports difficulty purchasing the quality-certified pediatric 3TC/ZDV due to low-volume purchase requests (G Arreghini, Médecins Sans Frontières, personal communication). Médecins Sans Frontières also notes difficulty obtaining the quality-certified pediatric ABC/3TC/ZDV, a new FDC not purchased by CHAI/UNITAID and therefore in low demand.

Even when pediatric ARVs are procured by large-scale purchasers like UNITAID, an unfortunate paradox comes into play in pediatric HIV/AIDS treatment: the more that pediatric ARV formulations are tailored to the needs of specific sub-groups, the less demand there is for a given product. This becomes particularly problematic in convincing companies to produce age-appropriate strengths of fixed-dose combination ARVs in multiple formulations. The WHO list of priority ARVs needs to be complete but also succinct, to aggregate demand around the most important products and avoid the development and production of ARVs that go unused by countries.

Because of the inherent disincentives for manufacturers in the pediatric ARV market, extreme care must be taken to ensure that price negotiations between producers and large-scale purchasers are conducted in a manner that ensures sufficient profit to sustain prices and stabilize the market over the long term. Activists, civil society organizations, researchers, international organizations and others must not only lobby for pediatric investments, but also monitor the movement of manufacturers in and out of the pediatric market, the extent and rate of new product uptake and their impact on child health. If countries are in a transition to new products, it is important for donors and suppliers to know and predict the amount of time needed for such transitions.

Operational research to identify and address reasons for low product utilization is a critical next step towards meeting two major global targets for 2015: Millennium Development Goal (MDG)-4, calling for a two-thirds reduction in mortality rates for children under five, and MDG-6, which aims to halt and begin to reverse the spread of HIV [46].

Success in the case of pediatric HIV/AIDS treatment cannot be determined only by market availability of new and improved ARV products. Until the barriers to uptake can be identified and addressed, and country demand stabilizes, organizations like UNITAID which offer the equivalent of advance market commitments will be needed to encourage the entry of new manufacturers and "hold the market" until countries can adopt the newer formulations.

### Limitations

While we systematically cleaned and validated purchases [30-32], it is possible that misreported purchases are still present in our analytic data set. The historical GFATM ARV transactional data posted in WHO *GPRM*, in particular, required considerable cleaning. We note a substantial number of ARV transactions in the "miscellaneous" category. Most of these miscellaneous transactions were reported by procurement agencies and we suspect many of these miscellaneous reports are actually purchases made by GFATM recipients. ARV transactions for GFATM recipients have been inconsistent in both the older GFATM *Purchase Price Report *and the WHO *GPRM*. We observed many instances over the past few years when transactions appeared and disappeared from both of these databases.

For this paper, we used 2002-2009 purchases downloaded from the WHO and GFATM on 1 May, 2010. We noted the absence of SCMS, UNITAID, and GFATM purchases in the WHO *GPRM *after April 2009 and therefore obtained this information directly from those organizations. The GFATM data was publicly available on its website. CHAI provided ARV purchase data to researchers on behalf of UNITAID without restrictions and SCMS staff provided transactional data to researchers under conditions that they review and comment on manuscripts utilizing their data prior to submission.

Recent interventions to improve the quality of transactions in the GFATM *PQR *have resulted in longer delays from the time countries report purchases to public posting. Our data therefore underestimate 2009 GFATM purchases. It is possible that GFATM-supported countries purchased new pediatric formulations in 2009 that do not yet appear in publicly posted data. In addition, some organizations (i.e., World Bank, PEPFAR purchases outside of SCMS, Médecins Sans Frontières) do not report ARV purchases to the WHO GPRM. Similarly, governments that purchase ARVs with their own funds do not report transactions to WHO. Delays in reporting, data restrictions imposed by some donors, and unwillingness to report ARV purchases will limit the ability to monitor and evaluate global ARV markets in a timely and unbiased manner.

We limited our analytic data set to ARV formulations used predominantly in children and infants. Our analyses did not include three ARVs (3TC150, EFV 600, and DRV) listed as pediatric ARVs by WHO (Table 1) as these are more commonly used for adults.

We calculated ARV regimen prices for new and liquid formulations using UNITAID/CHAI-reported prices because UNITAID was the only consistent purchaser of FDCs. These prices may not accurately reflect prices paid by countries outside of UNITAID programs. We acknowledge that some programs may still be splitting adult FDCs into quarters or crushing adult FDCs for use in children. We did not compare pediatric FDC prices to quarters of adult FDCs because WHO recommends against splitting adult tablets more than one time [33].

Liquid ARV prices are the most difficult to clean and validate given the multitude of different ways countries have misreported purchases. The Russian Federation accounted for more than 80% of GFATM brand purchases in 2008. It is possible that the Russian purchases were reported in error, but prior benchmarking price analysis of ARV purchases in the Former Soviet Union revealed that Russian prices (confirmed with procurement staff) were consistently and remarkably higher than other countries [47].

In addition, these Russian purchases passed through new quality improvement processes implemented when the new *PQR *system at the GFATM was recently established.

## Conclusion

Treatment of children with HIV/AIDS is a high priority for the international community. However, ensuring that needed pediatric medicines are developed and delivered to those who need them remains a complex, challenging task. In order to improve performance in this area, a better understanding of the pediatric ARV market is needed - where it is performing well, and where substantial market failures persist.

This study has demonstrated that the pediatric ARV market is not simply a smaller version of the adult market (described elsewhere [30-32]). Compared to HIV/AIDS treatment options for adults, far fewer ARVs have been proven safe and effective in children. Whereas multiple donors and countries buy substantial quantities of adult first-line ARVs, one international institution, UNITAID, plays a dominant role in the pediatric market, buying an overwhelming proportion of some pediatric ARVs. Pediatric markets become fragmented into niches with little demand as manufacturers develop more acceptable, age-appropriate pediatric products, and adopting improved formulations may present logistical challenges in some countries. While most adult FDCs are produced by several quality-certified manufacturers, many pediatric FDCs have only one quality-certified manufacturer, leaving HIV/AIDS treatment programs highly dependent on a single supplier to meet global demand.

Ensuring a long-term supply of high-quality, effective, affordable and well-adapted ARVs for children in different age groups will require ongoing monitoring and improved understanding of the global pediatric ARV market. Furthermore, much research is required at country level to understand better why uptake of new, improved formulations has been so slow, and what can be done to accelerate children's access to quality AIDS care in resource-poor settings. Continued innovation in pediatric ARV development may be threatened by outdated procurement practices failing to connect clinicians making prescribing decisions, supply chain staff dealing with logistics, donors, international organizations, and pharmaceutical manufacturers. Perceptions of global demand must be better informed by accurate estimates of actual country-level demand.

## Abbreviations

ABC: abacavir; ATV: atazanavir; DRV: darunavir; ddI: didanosine; EFV: efavirenz; FTC: emtricitabine; FPV: fosamprenavir; 3TC: lamivudine; LPV/r: lopinavir/ritonavir; NFV: nelfinavir; NVP: nevirapine; RTV: ritonavir; SQV: saquinavir; d4T: stavudine; ZDV: zidovudine.

## Competing interests

BW, TB and SM served as consultants for UNITAID. BW has accepted a position of employment at UNITAID.

## Authors' contributions

BW designed and coordinated the study, participated in data cleaning and data analysis, and was the lead author on this paper. ED, YL and MP contributed to data management, data analysis and editing of the manuscript. EJ, TB, and SM contributed to the writing of the manuscript and edited it for important content. All authors read and approved the final manuscript.

## Pre-publication history

The pre-publication history for this paper can be accessed here:

http://www.biomedcentral.com/1471-2431/10/74/prepub
